# A new approach to prostate cancer screening

**DOI:** 10.1515/almed-2023-0082

**Published:** 2023-08-03

**Authors:** Xavier Filella, Álvaro González, Josep Maria Augé, Antonio Barco, Rosa Carbonell, María Jesús Gaspar, Antonio Martínez-Peinado, Clara Pérez Barrios, Marta Sánchez-Carbayo, José Diego Santotoribio, Jaume Trapé, on behalf of the Comisión de Marcadores Biológicos de Cáncer, SEQCML

**Affiliations:** Service of Biochemistry and Molecular Genetics, CDB, Hospital Clínic de Barcelona, IDIBAPS, Barcelona, Spain; Service of Biochemistry, Clínica Universidad de Navarra, Pamplona, Spain; Service of Clinical Biochemistry, Miguel Servet University Hospital, Zaragoza, Spain; Laboratory of Biochemistry, San Juan University Hospital, San Juan, Alicante, Spain; Service of Clinical Analysis, Hospital Universitario Príncipe de Asturias, Alcalá de Henares, Madrid, Spain; Clinical Analsysis Management Unit, Section of Molecular Genetics, Reina Sofía University Hospital, Córdoba, Spain; Laboratory, Puerta de Hierro University Hospital, Majadahonda, Madrid, Spain; Department of Health Sciences, Universidad Pontificia, Salamanca, Spain; Laboratory, Hospital Universitario Virgen del Rocio, Seville, Spain; Laboratory, Althaia Xarxa Assitencial Universitària, Manresa, Spain

**Keywords:** prostate cancer, PSA, screening

## Abstract

Prostate cancer screening based on prostate-specific antigen (PSA) testing has been a matter of controversy. Although screening for prostate cancer was effective in reducing mortality, it resulted in overdiagnosis, which translated into unnecessary treatments and numerous adverse effects. As a result, recommendations from scientific societies became increasingly restrictive. In the recent years, new approaches to prostate cancer screening have been proposed. These new approaches are aimed at solving the controversy between widespread screening vs. no screening, and reconsidering PSA testing as a screening tool with a good benefit/risk balance. In this context, the European Association of Urology submitted a proposal to the European Commission for prostate cancer screening to be performed as a function of baseline PSA concentrations. The European Commission recently recommended the implementation of organized prostate cancer screening programs for men aged ≤70 years based on PSA values in combination with follow-up magnetic resonance imaging.

## Introduction

Prostate cancer (PCa) is the most common cancer in men in Spain, with 30,884 new cases diagnosed in 2022. Although five-year survival reaches 89.8 %, it caused 5,922 deaths in 2020 [1]. When early diagnosis is achieved, curative treatments improve prognosis.

Prostate cancer screening based on prostate-specific antigen (PSA) testing has been a subject of debate. The European Randomized Study of Screening for Prostate Cancer (ERSPC) revealed a 21 % reduction of PCa-related mortality in the screening group vs. the control group after 13-year follow-up [2]. The extended 16-year trial [3] uncovered reductions of 25 and 48 % in PCa-related mortality in men who underwent a single screening round vs. several screening rounds, respectively. This trial indicates that the number of men needed to avert a death decreased from 742 to 570 when follow-up was extended from 13 to 16 years.

The results of the ERSPC, however, are inconsistent with those obtained in the American PLCO clinical trial (prostate, lung, colorectal, and ovarian screening trial), where follow-up was extended to 17 years. The PCLO trial did not reveal any differences between the screening and the control group [4, 5]. In the same line, no differences were found either in a UK study involving 419,582 men aged 50–80 years [6].

These studies had two particularities. In the American trial, the rate of contamination by opportunistic PSA screening in the control arm reached 42–50 %; as the authors acknowledge, conclusive results could not be drawn about the usefulness of screening [7]. Secondly, the UK trial was based on a single PSA test round instead of several testing rounds over the years, as in the ERSPC and PLCO trials. In addition, the median follow-time in the UK study was only 10 years, and protocol adherence was as low as 40 %. As a result, the authors of the study recognize that a single PSA screening test may be inconclusive when evaluating the usefulness of PCa screening based on PSA.

In any case, widespread PSA testing led to significant overdiagnosis of indolent tumors. This phenomenon translated into unnecessary treatments and their associated adverse effects, ranging from urinary incontinence to impotence.

## Advantages and disadvantages of prostate cancer screening based on PSA testing

In 2012, in the light that the 13-year PLCO trial revealed a negative benefit/risk balance of PSA testing [4], the US Preventive Services Task Force (USPSTF) released a strong position statement against screening [8]. Since the publication of the PLCO study, scientific societies have released more restrictive recommendations about PSA-based PCa screening. As a result, the volume of PSA tests has decreased progressively. One of the five recommendations included in the Choosing Wisely initiative of the Internal Medicine Foundation in 2016 was not to perform PSA screening for PCa [9].

Consistently, the 2021 AEBM-ML document “*Decisiones inteligentes desde el laboratorio*: *de elegir sabiamente a no hacer*”, which updated the former version of 2015, stated that there is no conclusive evidence that PSA testing reduces mortality. The AEBM-ML recommended that the decision to undergo screening is made by the patient, who must be duly informed on the associated risks and benefits [10]. A similar position was communicated by the Spanish Society of Family and Community Medicine in 2017. This society recommended “*not to perform PSA testing routinely in asymptomatic patients without a first-degree familial history of prostate cancer*” [11]. The position of the Spanish Ministry of Health, Social Services and Equality is consistent with these recommendations. In April 2019, the Public Health Committee highlighted the negative benefit/risk balance of PSA testing for PCa screening [12].

Recommendations against PSA testing have resulted in delayed diagnosis of PCa. Bandini et al. [13] reported an increase in the incidence of new cases of metastatic PCa, based on an analysis of the SEER database (Surveillance, Epidemiology and End Results). The authors observed that, between 2004 and 2014, the age at diagnosis of metastatic PCa decreased, whereas the number of patients with metastatic disease at diagnosis increased from 1.9 to 2.4 per 100,000 inhabitants.

## Baseline PSA as a useful patient selection tool

In the recent years, several authors have proposed that PSA testing is reconsidered for PCa screening. A range of strategies – described as smart screening, personalized screening, or individualized screening – have been proposed to solve the controversy between widespread screening vs. no screening, and reconsider PSA testing as a screening tool with a good benefit/risk balance [14].

PSA testing has a poor specificity in patients with benign prostate diseases, often associated with prostate hyperplasia. In contrast, the specificity of PSA testing improves in young subjects, in whom prostate hyperplasia is less frequent. This is what explains the good predictive value of PSA for PCa screening reported by Stenmann et al. in 1994 [15]. The authors documented that a PSA increase >2.5 μg/L was predictive of a diagnosis of PCa in the following decade.

Subsequent studies confirmed this finding, although it has not been until recently that data from a large cohort has been provided. This evidence was obtained from a secondary analysis of a cohort from the American 13-year PLCO trial, which included 10,968 men aged 55–60 years. The study revealed an association between baseline PSA concentrations and future diagnosis of clinically significant PCa. According to the authors, the frequency of PSA testing can be reduced in men with baseline PSA <2 μg/L and discontinued when baseline PSA levels are <1 μg/L [16].

Personalized PCa screening based on baseline PSA would improve screening results. Hence, this strategy would reduce overdiagnosis resulting from false positives in older men related to the presence of benign prostate diseases. This strategy would also involve cost savings, since PSA testing would not be performed in numerous patients with a low baseline PSA, in the absence of other significant evidence.

## New prostate cancer screening tests

In the last years, the scientific community has witnessed a significant evolution in PCa screening strategies. Firstly, active surveillance has been validated as an effective attitude in PCa patients with a low probability of progression. In these cases, treatment can be delayed until it is required due to a change in the characteristics of the tumor [17]. Secondly, the risk for PCa can be assessed by multiparametric magnetic resonance (mpMRI), which facilitates clinical decision-making about biopsy. In this sense, Göteborg 2 [18] highlights the capacity of mpMRI to detect clinically significant tumors, thereby reducing overdiagnosis. Finally, there is evidence of the usefulness of new biomarkers such as 4kscore (algorithm combining total, free and intact PSA, and kallikrein 2, when considered in relation to the age of the patient, the result of digital rectal examination and potential availability of a previous negative biopsy). Other potential markers include the Prostate Health Index (PHI, which combines total and free PSA and [−2]proPSA); or Stockholm 3 test (S3M, which combines five proteins; total and free PSA; kallikrein 2; MSMB and MIC1, in combination with a panel of 254 CaP-related SNPs, and demographic and clinical data including familial history and previous prostate biopsies), which have a higher specificity than PSA and are associated with higher tumor aggressiveness, thereby reducing overdiagnosis [19].

Of special interest is the 4kscore test, which is used to specifically detect high-grade PCa (Gleason ≥7). An American multicenter study involving 1,012 men demonstrate that the 4kscore test reduces the number of biopsies by 30 %, whereas diagnosis is delayed in only 1.3 % of patients, with an area under the curve of 0.821 [20]. Additionally, baseline 4kscore results enable the stratification of patients based on their long-term risk of developing metastatic PCa, with its effectiveness being superior to that of PSA testing [21].

PHI is associated with tumor aggressiveness, as demonstrated by Maxeiner et al. [22]. The authors observed an association between PHI and the probability of biochemical relapse in a series of 437 patients treated with radical prostatectomy. The specificity of PHI is superior to that of PSA and free PSA rate in detecting PCa and PCa with an intermediate/high risk of progression. This index is especially effective in patients with a small (area under the curve for PHI of 0.817) or medium (area under the curve for PHI of 0.759) prostate [23]. Young patients, who have a smaller prostate, are the population that may benefit the most from the use of PHI. Interestingly, Druskin et al. [24] documented that the area under the curve increased from 0.83 to 0.90 when PHI density, calculated as the relation between PHI and prostate volume in mL, was included in a baseline model that considered age, previous negative biopsy, and mpMRI results.

S3M has also shown a high specificity and strong association with tumor aggressiveness. The screening study carried out by Stavanger (Norway) [25] revealed an increase in the detection of cases of PCa with a Gleason degree ≥7 and a decrease in the detection of clinically non-significant tumors when S3M testing was performed instead of PSA testing.

## Controversy raised by the European Association of Urology’s proposal

The aim of any PCa screening scheme should be to reduce the number of unnecessary biopsies, prevent overdiagnosis, and improve diagnosis of clinically significant tumors. The goal is to reduce cancer-related mortality and decrease the number of cases of metastatic PCa at diagnosis.

The commutability of results from different PSA assays would help reduce ineffective practices and validate the quality of screening schemes. It would also prevent errors in patient selection for biopsy when a standard cut-off point is used for assays measuring different PSA concentrations. The international standard established by the WHO in 1999 helped reduce variability across assays. However, inconsistencies persist across assays when measuring PSA concentrations [26], [27], [28]. These differences are not always reported in clinical guidelines [29]. Manufacturers should make additional efforts to standardize PSA assays and establish universally acceptable limits for therapeutic decision-making.

In 2017, the European Association of Urology (EAU) recommended to reconsider PCa screening approach according to baseline PSA [30], [31], [32]. Additionally, the European Commission recently released an initiative to intensify cancer screening based on the data currently available, to improve early detection and prognosis. In a document dated 2022 September 20th, European countries are recommended to use organized PCa screening programs for men aged ≤70 years on the basis of their PSA concentrations, in combination with follow-up magnetic resonance imaging [33, 34]. To such purpose, the particularities of each member state should be considered, in terms of the technology available and incidence of PCa in each country. The EU4-Health programme recently approved Prostate cancer Awareness and Initiative Screening in the European Union (Praise-U) [35]. Praise-U is a three-year project involving a multidisciplinary team composed of researchers from 25 institutions from 12 member states. Its mission is to reduce morbidity and mortality caused by PCa while avoiding overdiagnosis. This strategy is based on a nationally-tailored algorithm for early detection.

## Conclusions

The results available suggest that baseline PSA values may be useful in identifying the patients that will benefit the most from inclusion in screening programmes, while avoiding potential prejudices to other patients. The use of mpMRI in PCa screening has resulted in significant improvements and a reduction of overdiagnosis, thereby increasing the effectiveness of screening programs. Further, prospective, multicenter studies are necessary to confirm that new biomarkers are superior to PSA testing, in terms of specificity and potential capacity to select patients at a higher risk of developing aggressive PCa. Such is the case of the 4kscore, which may be useful for selecting patients with a high risk for progression into aggressive PCa. In the light of the evidence available, and in accordance with the European Commission guidelines, it is necessary to reconsider recommendations from scientific societies and health policies on PCa screening and, more specifically, PSA-based PCa screening.
